# Comparative Analysis of Visual Outcomes and Complications in Intraocular Collamer Lens, Small-Incision Lenticule Extraction, and Laser-Assisted In Situ Keratomileusis Surgeries: A Comprehensive Review

**DOI:** 10.7759/cureus.58718

**Published:** 2024-04-22

**Authors:** Uma Swaminathan, Sachin Daigavane

**Affiliations:** 1 Ophthalmology, Jawaharlal Nehru Medical College, Datta Meghe Institute of Higher Education & Research, Wardha, IND

**Keywords:** small-incision lenticule extraction (smile), complications, visual outcomes, laser-assisted in situ keratomileusis (lasik), intraocular collamer lens (icl), refractive surgery

## Abstract

This review provides a comparative analysis of visual outcomes and complications associated with three prominent refractive surgical techniques: intraocular collamer lens (ICL) implantation, small-incision lenticule extraction (SMILE), and laser-assisted in situ keratomileusis (LASIK). Refractive surgeries aim to correct myopia, hyperopia, and astigmatism, offering patients an alternative to corrective lenses. The review highlights the importance of comparing these procedures to guide clinical decision-making effectively. Each technique is described, emphasizing its unique advantages and considerations. While LASIK remains widely favored for its rapid visual recovery and high patient satisfaction, ICL is suitable for patients with higher refractive errors or corneal irregularities. Although relatively newer, SMILE shows promise with potential benefits such as corneal biomechanical stability and a reduced risk of dry eye syndrome. However, each procedure carries its distinct complications, reinforcing the need for personalized patient care and informed decision-making. Understanding these techniques' relative efficacy and safety profiles is essential for optimizing outcomes and enhancing patient satisfaction. Continued advancements in technology and surgical techniques promise further improvements in refractive surgery outcomes, underscoring the importance of ongoing research and innovation.

## Introduction and background

Refractive surgeries encompass a range of procedures to correct various vision impairments, most commonly myopia (nearsightedness), hyperopia (farsightedness), and astigmatism. These procedures aim to alter the cornea's shape or the eye's focusing properties to improve visual acuity without needing corrective lenses. Over the years, technological advancements and surgical techniques have led to several effective procedures, each with unique benefits and considerations [1]. Given the diversity of available refractive surgical techniques, comparing their visual outcomes and associated complications is crucial. Such comparative analyses provide valuable insights for clinicians, helping them make informed decisions about the most suitable procedure for individual patients based on their specific visual needs, ocular characteristics, and risk profiles. Moreover, understanding different procedures' relative efficacy and safety profiles contributes to advancing clinical practice and enhancing patient outcomes [2].

Intraocular collamer lens (ICL) implantation, small-incision lenticule extraction (SMILE), and laser-assisted in situ keratomileusis (LASIK) offer distinct advantages and considerations, catering to patients' diverse needs and preferences seeking vision correction. ICL involves the insertion of a phakic intraocular lens (IOL) into the eye's posterior chamber, typically in front of the natural lens. This procedure suits patients with moderate-to-high refractive errors or corneas unsuitable for laser-based surgeries [3]. SMILE is a minimally invasive refractive surgery that involves the creation of a corneal lenticule, which is then removed through a small incision, reshaping the cornea and correcting refractive errors. SMILE offers potential advantages over LASIK, including preservation of corneal biomechanical integrity and a reduced risk of dry eye syndrome [4]. LASIK is one of the most widely performed refractive surgeries worldwide. It involves creating a corneal flap and applying an excimer laser to reshape the underlying corneal stroma, correcting refractive errors. LASIK is known for its rapid visual recovery and high patient satisfaction rates [1].

The primary objective of this review is to provide a comprehensive comparative analysis of the visual outcomes and complications associated with ICL, SMILE, and LASIK surgeries. By synthesizing existing literature and clinical evidence, we aim to offer insights into these procedures' relative efficacy, safety, and suitability for different patient populations. Additionally, we seek to identify critical factors influencing surgical outcomes and discuss the implications of our findings for clinical practice and future research directions.

## Review

ICL surgery

Procedure Overview

ICL surgery is designed to address refractive errors by implanting a collamer lens into the eye. The collamer lens, composed of a collagen material known as collamer, combines collagen with a polymeric material that possesses flexible properties akin to the collagen in the cornea. Conducted by a specially trained eye surgeon, this outpatient procedure typically takes place in a sterile operating room to mitigate infection risks [5,6]. The ICL is permanently inserted throughout the surgery between the eye's natural lens and the iris, collaborating with the natural lens to refract light onto the retina, enhancing visual clarity. The ICL does not alter the eye's structure but augments its focusing capability. This surgery is suitable for individuals who may not meet the criteria for LASIK surgery and can effectively address higher degrees of nearsightedness. Moreover, it is reversible, as the ICL can be extracted if necessary [6,7]. The advantages of ICL surgery encompass the potential for enhanced vision without reliance on glasses or contact lenses. Research suggests that nearly 95% of individuals undergoing this procedure report satisfaction or high satisfaction with the outcomes. Nevertheless, like any surgical intervention, ICL surgery entails inherent risks and possible complications, including endothelial damage, cataract development, heightened intraocular pressure, and the potential for under- or overcorrection [7,8]. The financial aspect of ICL surgery may vary, warranting individuals to consult their insurance provider regarding coverage. The estimated average cost of ICL surgery is approximately $4,000 per eye, yet this figure may fluctuate depending on geographical location and the surgeon's fees. Furthermore, it is imperative to undergo a comprehensive eye examination and evaluation to ascertain suitability for ICL surgery [5,7].

Visual Outcomes

ICL surgery involves permanently placing a collamer lens between the eye's natural lens and iris to correct refractive errors. By working with the natural lens, the ICL aids in refracting light onto the retina, improving visual clarity. Typically conducted as an outpatient procedure within a sterile operating environment to minimize infection risks, ICL surgery offers a viable option for individuals who may not qualify for LASIK and can effectively address a higher degree of nearsightedness. While generally safe, the surgery presents potential risks, including cataract formation and increased intraocular pressure, necessitating thorough preoperative evaluation and postoperative care. Despite these considerations, ICL surgery presents benefits such as reduced dependence on corrective eyewear and enhanced night vision. However, its cost may vary, requiring individuals to verify coverage with their insurance provider [5,6,8]. Studies have demonstrated favorable visual outcomes associated with ICL surgery, with high patient satisfaction rates and notable improvements in uncorrected visual acuity. ICL implantation has been shown to induce fewer ocular higher-order aberrations than alternative procedures, offering better vision and life outcomes than LASIK. Furthermore, the surgery is linked with a low incidence of significant cataract formation and excellent visual quality, particularly for individuals with high refractive prescriptions. Despite these benefits, it is crucial to consider potential risks and limitations, including careful patient selection, the possibility of postoperative visual disturbances, and the importance of ongoing monitoring and management of complications [8-10].

Complications and Their Management

Complications associated with ICL surgery encompass both intraoperative and postoperative challenges. Common issues are abnormal arch height, malpositioning of the ICL, loss of corneal endothelial cells, corneal decompensation, elevated intraocular pressure, secondary glaucoma, cataract formation, and night vision disturbances [5,11]. Studies have indicated that complications such as endothelial cell loss, corneal decompensation, elevated intraocular pressure, and secondary glaucoma tend to be relatively lower in ICLs equipped with a central hole than those without it. However, postoperative complications such as night vision disturbances may be more pronounced in ICLs lacking a central pore [11]. Surgical trauma during ICL implantation can lead to a range of complications, including conjunctival or intraocular hemorrhage, corneal epithelial defects, corneal edema, and traumatic cataract formation [11]. Managing these complications typically involves employing specific surgical techniques, meticulous postoperative care, and, in certain instances, additional procedures. It is imperative for individuals considering ICL surgery to familiarize themselves with these potential complications and engage in thorough discussions with their eye care provider to ensure informed decision-making.

Comparative Analysis With SMILE and LASIK

Several research articles have conducted comparative analyses between SMILE and LASIK surgeries, shedding light on their outcomes and complications. One such study, focusing on patients with myopia, revealed that both SMILE and LASIK were safe and effective procedures. However, SMILE exhibited a lower incidence of dry eye symptoms and yielded superior visual outcomes during the early postoperative period [12,13]. Similarly, another study examining patients with high myopia corroborated these findings, indicating that SMILE and LASIK were safe and effective. SMILE again demonstrated a lower occurrence of dry eye symptoms and superior visual outcomes shortly after surgery [12,13]. A meta-analysis encompassing multiple studies comparing SMILE and LASIK reiterated these trends, highlighting the safety and efficacy of both procedures while emphasizing SMILE's advantages in terms of reduced dry eye symptoms and improved early postoperative visual outcomes [14]. Ultimately, the decision between SMILE and LASIK hinges on individual factors, necessitating patients to consult their eye care provider to determine the most suitable procedure for their needs [15].

SMILE surgery

Procedure Overview

SMILE represents a minimally invasive refractive surgery technique utilizing femtosecond laser technology to create a corneal lenticule, subsequently extracted through a small incision. Targeting myopia, hyperopia, and astigmatism, SMILE offers notable advantages over other refractive procedures, including expedited recovery, minimized corneal damage, and potential biomechanical benefits. The procedure entails forming lower and upper lenticule interfaces and a 2-3 mm incision for lenticule extraction. FDA-approved for myopia correction, SMILE stands as a cost-effective alternative to LASIK, requiring only a single laser platform [16,17]. SMILE demonstrates comparable safety, efficacy, and predictability outcomes to LASIK, with heightened patient satisfaction and diminished postoperative dry eye incidence. Nevertheless, SMILE presents limitations, notably a heightened risk of under-correction and regression in higher myopic corrections and astigmatism cases [18]. Since its inception in 2007, SMILE has garnered widespread adoption, with over 5 million procedures performed globally. While introduced to the U.S. market in 2016, SMILE attained approval elsewhere approximately a decade earlier [18].

Visual Outcomes

The visual outcomes of SMILE generally parallel those of LASIK, characterized by exceptional postoperative visual acuity and refractive outcomes [19-21]. A comparative study between SMILE and LASIK revealed that approximately 90%-95% of patients achieved uncorrected distance visual acuity of 20/20 within the initial day following SMILE surgery [21]. SMILE's visual recovery process is notably swifter than LASIK [21]. Studies examining SMILE in patients with thin corneas have demonstrated its safety and efficacy in treating myopia, yielding satisfactory outcomes [21,22]. However, visual outcomes in individuals with thin corneas may lag slightly behind those with average corneal thickness [22]. Despite this, SMILE consistently delivers excellent visual outcomes akin to LASIK, boasting expedited visual recovery and a reduced risk of postoperative dry eye. Nevertheless, it is imperative to note that SMILE may not yield optimal results in patients with thin corneas, necessitating alternative approaches to nomogram calculation for this patient demographic [22].

Complications and Their Management

SMILE surgery, while generally safe and effective, carries potential complications, as with any surgical procedure. Among the complications associated with SMILE surgery are bulging, blindness, night vision issues, debris, infection, and miscorrection [23]. Bulging, albeit rare, can occur if the eye weakens post-surgery, potentially resulting in vision disturbances. Blindness or partial loss of sight, although exceedingly rare, remains a possible complication inherent to any eye operation. Night vision issues such as glare and halos may manifest following SMILE surgery, albeit transiently, and can typically be managed with night lenses or other remedial measures. Debris may arise from removing the corneal disc during the procedure, leading to irritation and inflammation, though such occurrences can generally be alleviated through non-invasive treatments. While infection is a risk associated with any surgical intervention, the likelihood of infection with SMILE surgery is relatively low. Miscorrection, characterized by over or under-correction of vision problems, may necessitate retreatment. The success of SMILE surgery is often contingent upon the experience and skill of the surgeon, with complications typically managed through appropriate techniques [23].

Comparative Analysis With ICL and LASIK

SMILE surgery has been subject to comparative analyses against LASIK and ICL procedures regarding visual outcomes and complications, revealing notable insights. SMILE demonstrates comparable refractive correction capabilities to LASIK, alongside potential advantages such as expedited recovery from postoperative dry eye, accelerated reinnervation of corneal nerves, and biomechanical benefits [24]. A meta-analysis revealed no significant disparities between SMILE and LASIK in critical parameters, including refractive spherical equivalent, loss of corrected vision lines, attainment of 20/20 uncorrected distance visual acuity, and proximity to target spherical equivalent [24]. Moreover, SMILE is associated with heightened patient satisfaction and reduced postoperative dry eye incidence relative to LASIK [24]. Notably, SMILE's cost-effectiveness stems from its requirement for a single laser platform compared to LASIK's dual-platform setup [24]. Demonstrating safety and efficacy across myopia, hyperopia, and astigmatism, SMILE mirrors LASIK in terms of efficacy and predictability outcomes [25]. However, comparisons with ICL indicate ICL's superiority in safety and efficacy for patients with higher myopic degrees [25]. Intraoperative complications for SMILE include corneal cap perforation, incisional tear, lenticule dissection difficulties, lenticule remnant, bleeding, and partial centering [19]. However, advancements in surgical proficiency and the widespread adoption of SMILE technology have mitigated such complications [19].

LASIK surgery

Procedure Overview

LASIK represents a prevalent ophthalmologic surgical intervention utilized to correct refractive errors, aiming to diminish an individual's reliance on glasses or contact lenses through permanent alteration of corneal shape using an excimer laser. The procedure involves creating a flap in the cornea, followed by a computer-controlled laser application to reshape the cornea and rectify ocular refraction issues. Completed typically within 30 minutes or less, LASIK is renowned for its swift visual recovery and minimal patient discomfort. It is one of the most successful refractive surgeries, with over 99% patient satisfaction rates [26-28]. Employing wavefront-guided technology for meticulous eye measurements, LASIK ensures precise tissue removal. Preceding the surgery, a comprehensive preoperative assessment is conducted to elucidate the procedure's risks and benefits and address patient queries. However, LASIK is contraindicated for individuals with specific eye conditions such as autoimmune disorders, persistent dry eyes, or certain corneal disorders [29]. Acknowledged for its safety and effectiveness in rectifying refractive errors, LASIK remains associated with inherent risks akin to any surgical intervention. Potential complications include dry eyes, flap-related issues, and the risk of haze or scarring. The selection between LASIK and comparable procedures, such as laser-assisted sub-epithelial keratectomy (LASEK), hinges on individual suitability and preferences [30].

Visual Outcomes

Visual outcomes following LASIK are typically outstanding, with most patients achieving near-optimal vision. A comparative study between LASIK and photorefractive keratectomy (PRK) showed that LASIK patients exhibited a higher satisfaction rate, although PRK demonstrated a superior efficacy index and predictability [31]. Utilization of wavefront-guided LASIK has shown enhancements in outcomes, particularly in contrast sensitivity, night vision, and alleviation of visual symptoms [28]. In a retrospective analysis comparing LASIK and femtosecond LASIK (FS-LASIK), FS-LASIK demonstrated superior visual outcomes, albeit patients reported higher satisfaction with PRK [31]. LASIK tends to be more efficacious in individuals with lower degrees of myopia and astigmatism. At the same time, it may be less suitable for those with thin corneas, dry eyes, or contact sports [12]. LASIK yields exceptional visual outcomes for most patients, with specific procedures such as PRK boasting higher satisfaction rates. Nonetheless, LASIK's effectiveness tends to be more pronounced for lower degrees of myopia and astigmatism, with considerations required for individuals with thin corneas, dry eyes, or involved in contact sports.

Complications and Their Management

Complications arising from LASIK surgery can be classified into intraoperative and postoperative categories [32]. Intraoperative complications encompass a range of issues, such as suction loss, free cap formation, flap tears, buttonhole flaps, decentered ablation, central islands, interface debris, and femtosecond laser-related complications [33]. On the other hand, postoperative complications may include flap striae, flap dislocation, residual refractive errors, diffuse lamellar keratitis, microbial keratitis, epithelial ingrowth, overcorrection, under-correction, visual aberrations, rainbow glare, corneal epithelial defects, ectasia, and loss of best spectacle-corrected visual acuity [32,33]. Dry eyes are the most prevalent postoperative complication, affecting 60%-70% of patients [32]. Management strategies for these complications vary based on their type and severity. For instance, flap dislocation can often be addressed by repositioning the flap, suturing it in persistent folds, and employing lubricants to promote healing [32]. Conversely, infections beneath the flap necessitate immediate intervention, including flap lift and irrigation, culture analysis, and administration of broad-spectrum topical antibiotics [34]. Preventative measures to mitigate complications involve meticulous preoperative screening, management of treatable retinal lesions, and comprehensive patient counseling [33]. If experiencing complications following LASIK surgery, patients are advised to consult their eye care provider promptly to determine the most appropriate course of action.

Comparative Analysis With ICL and SMILE

Several studies have conducted comparative analyses of ICL and SMILE procedures, revealing valuable insights. In one study, the visual quality of ICL and SMILE procedures was compared, with findings indicating that ICL implantation demonstrated a significantly higher safety index than SMILE. At the same time, SMILE exhibited a slightly superior predictability profile [10]. Similarly, another study focused on outcomes for high myopia, revealing that ICL implantation resulted in better refractive accuracy, improved uncorrected distance visual acuity, fewer higher-order aberrations, and enhanced subjective quality of vision compared to SMILE [35]. A meta-analysis examining the visual quality of ICL and SMILE procedures reinforced these trends, with postoperative visual quality reported to be marginally superior for ICL implantation compared to SMILE. Additionally, ICL was associated with lower induction of higher-order aberrations relative to SMILE. However, SMILE demonstrated a slightly superior predictability profile in this analysis [10]. Ultimately, the choice between ICL and SMILE hinges on individual factors, underscoring the importance of patients consulting with their eye care provider to determine the most suitable procedure for their needs [36].

Comparative analysis

Visual Outcomes Comparison Between ICL, SMILE, and LASIK

Various studies have undertaken comparative analyses of the visual outcomes and complications associated with ICL, SMILE, and LASIK surgeries, yielding insightful findings. One study comparing these procedures revealed no significant differences in postoperative visual outcomes, safety, and efficacy indices among SMILE, FS-LASIK, and ICL, highlighting their comparable performance [37]. Conversely, a meta-analysis examining visual outcomes, optical quality, and aberrations of SMILE and ICL indicated that ICL exhibited superior safety, efficacy, predictability, and stability compared to LASIK [38]. Moreover, a retrospective study underscored ICL's safety and efficacy in high myopia cases, demonstrating comparable long-term visual stability and patient satisfaction for low myopia correction [39]. Additionally, a comparative analysis between ICL and LASIK accentuated ICL's efficacy for individuals with higher myopic degrees, contrasting LASIK's affordability and shorter recovery period [14]. Ultimately, selecting these procedures hinges on individual needs, necessitating patients to consult their eye care provider to ascertain the most suitable option.

Factors Influencing Outcomes and Complications

Several studies have delved into the comparative analysis of visual outcomes and complications associated with different vision correction surgeries, catering to varying degrees of myopia and employing different surgical techniques. One such meta-analysis scrutinized disparities in visual outcome and optical quality between SMILE and ICL surgeries. The study assessed potential visual outcomes and optical quality variations between the two procedures, shedding light on their comparative efficacy [38]. Another study delved into the dynamic changes and influencing factors of visual symptoms following ICL and LASIK surgeries. With a focus on understanding the factors influencing visual symptoms post-surgery, this research sought to uncover the dynamic changes occurring and the factors contributing to visual symptoms following these procedures [42]. A separate investigation compared the visual outcomes after SMILE and ICL V4c surgeries for moderate myopia, presenting 1-year results to provide insights into the visual outcomes of these two procedures. By assessing the visual outcomes of SMILE and ICL V4c surgeries specifically for moderate myopia, this study aimed to offer valuable insights into their comparative efficacy and safety [43]. Furthermore, a comparative study scrutinized the indications and outcomes of surgery for high myopia, explicitly focusing on the STAAR ICL and LASIK. This research aimed to assess the safety and effectiveness of ICL versus LASIK for patients with high myopia, providing crucial insights into the suitability of each procedure for this demographic [41]. The findings in comparing the ICL and LASIK for low myopia revealed the statistically superior predictability and safety of ICL over LASIK. This study aimed to evaluate the visual outcomes and complications of these two procedures for low myopia, offering valuable insights into their respective efficacy and safety profiles [12].

## Conclusions

In conclusion, our comprehensive review and comparative analysis shed light on the visual outcomes and complications associated with ICL, SMILE, and LASIK surgeries. Across all three procedures, significant efficacy in correcting refractive errors and enhancing visual acuity was evident, albeit with variations based on individual patient characteristics. LASIK emerged as a widely favored option, offering rapid visual recovery and high patient satisfaction rates. At the same time, ICL implantation provided a viable alternative for individuals with higher refractive errors or corneal irregularities. Although relatively newer, SMILE surgery demonstrated promising results, with potential advantages in terms of corneal biomechanical stability and reduced risk of dry eye syndrome compared to LASIK. While each technique carries unique risks, the overall assessment underscores the importance of personalized patient care and shared decision-making in refractive surgery. The selection of the most suitable procedure should consider factors such as refractive error magnitude, corneal characteristics, ocular health status, and patient preferences. Continued advancements in technology and surgical techniques hold promise for further improving outcomes and reducing complications in refractive surgery, emphasizing the need for ongoing research and innovation in this critical area of ophthalmic care.
